# High-risk lesions of the breast: concurrent diagnostic tools and management recommendations

**DOI:** 10.1186/s13244-021-01005-6

**Published:** 2021-05-26

**Authors:** Francesca Catanzariti, Daly Avendano, Giuseppe Cicero, Margarita Garza-Montemayor, Carmelo Sofia, Emmanuele Venanzi Rullo, Giorgio Ascenti, Katja Pinker-Domenig, Maria Adele Marino

**Affiliations:** 1 Department of Biomedical Sciences and Morphologic and Functional Imaging, University of Messina, Messina, Italy; 2 Department of Breast Imaging, Breast Cancer Center TecSalud, ITESM Monterrey, Nuevo Leon, Mexico; 3 Unit of Infectious Diseases, Department of Clinical and Experimental Medicine, University of Messina, 98124 Messina, Italy; 4 Department of Radiology, Breast Imaging Service, Memorial Sloan Kettering Cancer Center, Suite 705, 300 E 66th Street, New York, NY 10065 USA; 5 Department of Biomedical Imaging and Image-Guided Therapy, Medical University of Vienna, Vienna, Austria

**Keywords:** High-risk lesions, Ultrasound, Mammography, B3 lesions, Breast imaging

## Abstract

Breast lesions with uncertain malignant behavior, also known as high-risk or B3 lesions, are composed of a variety of pathologies with differing risks of associated malignancy. While open excision was previously preferred to manage all high-risk lesions, tailored management has been increasingly favored to reduce overtreatment and spare patients from unnecessary anxiety or high healthcare costs associated with surgical excision. The purpose of this work is to provide the reader with an accurate overview focused on the main high-risk lesions of the breast: atypical intraductal epithelial proliferation (atypical ductal hyperplasia), lobular neoplasia (including the subcategories lobular carcinoma in situ and atypical lobular hyperplasia), flat epithelial atypia, radial scar and papillary lesions, and phyllodes tumor. Beyond merely presenting the radiological aspects of these lesions and the recent literature, information about their potential upgrade rates is discussed in order to provide a useful guide for appropriate clinical management while avoiding the risks of unnecessary surgical intervention (overtreatment).

## Key points

- B3 lesions are various pathologies with differing risks of associated malignancy.
- Tailored management of B3 lesions has been increasingly favored to reduce overtreatment.
- Most patients with high-risk lesions may be sparred surgical excision.

## Background

Breast lesions with uncertain malignant behavior, also known as high-risk or B3 lesions, are composed of a variety of pathologies with differing risks of associated malignancy. In the past several decades, the diagnosis of B3 lesions has significantly increased with the widespread implementation of screening programs and the advent of new imaging modalities [1]. B3 lesions reportedly represent approximately 3–21% of all breast lesions and carry a low but potential risk of associated malignancy ranging from 0.2 to 5% [2, 3]. B3 lesions are considered non-obligate precursors of malignancy because they can develop into higher-grade lesions, predominantly ductal carcinoma in situ (DCIS) and less frequently low-grade invasive tumors [4–6]. Some B3 lesions also function as risk indicators; for example, women diagnosed with a proliferative breast disease with atypia have a fourfold to tenfold higher risk of developing breast cancer compared with those without atypia [7, 8] in any location of the same or the contralateral breast [9].

Overall, high-risk lesions have a low risk of malignancy. Nevertheless, up to 35% of lesions of uncertain malignant potential (B3) are subsequently upgraded at surgical specimen to DCIS or low-grade carcinoma [5]. These lesions are highly heterogeneous and can be classified according to the presence or absence of associated atypia. For this reason, some authors suggested a further histological classification for B3 lesions into B3a and B3b depending on whether atypia is absent or present [10]. While open excision was previously preferred to manage all high-risk lesions, tailored management has been increasingly favored to reduce overtreatment and spare patients from unnecessary anxiety or high healthcare costs associated with surgical excision. According to recent guidelines, high-risk lesions may be managed with imaging follow-up, vacuum-assisted excision (VAE) or open excision [5, 11]. In some particular cases such as benign papillomas, VAE is used to replace surgical diagnostic biopsy. Compared to VAB, the sampling in VAE is performed with larger needles (7 gauge to 11 gauge needles) and about 4 g of tissue is removed, which is often estimated by multiplying the number of cores with estimated weight of each core dependent on the needle size. This procedure largely reduces the risk of false-negative results or pathological underestimation [11]. For most patients with small high-risk lesions (< than 20 mm), excision with VAE ensures complete sampling and might be sufficient as the only therapeutic method [12–14]. For patients with atypical intraductal epithelial proliferation, papilloma with atypia or phyllodes tumor, surgical management is still preferred. As to the time of follow-up and the imaging method to be used in patients with a personal history of a B3 lesion, there is still a lack of evidence to establish comprehensive guidelines.

The purpose of this work is to provide the reader with an accurate overview focused on the main high-risk lesions of the breast: atypical intraductal epithelial proliferation (AIEDP), lobular neoplasia (including the subcategories lobular carcinoma in situ and atypical lobular hyperplasia), flat epithelial atypia, radial scar and papillary lesions, and phyllodes tumor. Beyond presenting the radiological aspects of these lesions and the recent literature, information about their potential upgrade rates will be discussed in order to provide useful guidance for appropriate clinical management while avoiding the risks of unnecessary surgical intervention (overtreatment). In Table 1, the management and radiological follow-up recommendations for each high-risk lesion have been summarized. We note that further studies are needed to enable a more personalized approach; new diagnostic tools such as artificial intelligence could find their place in tailoring the diagnostic process and determine the appropriate imaging follow-up.

**Table 1:** Consensus recommendations for the management of B3 lesions

High-risk lesion	Management
Lobular Neoplasia (LN)	Surgical excision or VAE If after VAB, the lesion has been radiologically removed, imaging follow-up is recommended
Atypical Intraductal Epithelial Proliferation (AIDEP)	Surgical excision or VAE VAE is suggested in unifocal ADH in small lesions If the lesion has been removed completely and only focal ADH with calcifications exists, imaging follow-up recommended
Flat Epithelial Atypia (FEA)	VAE If after VAB, the lesion has been radiologically removed, imaging follow-up
Papillary Lesion (PL)	
With atypia	Surgical excision and imaging follow-up
Without atypia	VAE
Phyllodes Tumor (PT)	Surgical Excision
Radial Scar (RS)	
With atypia	VAE and imaging follow-up
Without atypia	VAE

Modified from Rageth CJ et al. Breast Cancer Res Treat (2018) [5] and reprinted under a Creative Commons Attribution 4.0 International License

## Atypical ductal hyperplasia

Atypical ductal hyperplasia (ADH) is one of the most common B3 lesions of the breast and the one with the highest risk of malignant transformation. It is therefore considered a non-obligate precursor of invasive cancer as well as an independent risk factor for breast cancer [15, 16]. ADH represents an intraductal proliferative lesion similar to usual ductal hyperplasia and low-grade ductal carcinoma in situ (DCIS), with features of low-grade atypia such as monomorphic nuclei with clear membranous borders and secondary intraluminal adenoid architecture.

ADH is diagnosed in 12–17% of percutaneous biopsies [17] in patients from a wide age range, from adolescence to old age [18]. ADH is defined by atypical epithelial proliferation restricted to one terminal ductal-lobular unit (TDLU) of ≤ 2 mm in maximal extension. Of note, the differential diagnosis between ADH and low-grade DCIS is based only on lesion size. As such, ADH cannot be definitively diagnosed over low-grade DCIS at percutaneous biopsy, as the biopsied sample may belong to a larger low-grade DCIS lesion that was not entirely sampled; instead, some authors recommend use the term AIDEP to describe findings indicating ADH or low-grade DCIS at percutaneous biopsy [6, 10, 19].

Upgrade rates to DCIS or invasive carcinoma from core needle biopsy to excision in the literature range from 0 to 62% for ADH [20]. The reported average upgrade rates for ADH are: 44% for 14-gauge spring-loaded stereotactic core biopsy, 24% for 14-gauge directional VAB, and 19% for 11-gauge VAB needles [21]. In Table 2, upgrade rates for ADH as well as for the other B3 lesions are summarized.

**Table 2:** Upgrade rates for high-risk lesions [5, 15, 22–26]

High-risk lesion	Upgrade rate to malignancy	Upgrade Rate to Malignancy by Core-Needle Biopsy (CNB) or Vacuum-Assisted Biopsy (VAB)
Lobular neoplasia (LN)	17% (95% CI 13–21%)	Overall range 0–40%
Atypical lobular hyperplasia (ALH)	12% (95% CI 5–21%)	CNB (14G) 16% VAB (9G): 6–25.4% for ALH and LCIS
Lobular carcinoma in situ (LCIS)	22% (95% CI 14–31%)	CNB (14G) 25% VAB: 6–25.4% for ALH and LCIS
Atypical Intraductal Epithelial Proliferation (AIDEP)	28% (95% CI 24–31%)	CNB (14G) 31% VAB (14G) 24% & (11G) 19%
Flat epithelial atypia (FEA)	11% (95% CI 8–14%)	CNB (14G) 10–21% VAB (11G) 6%
Papillary lesion (PL)	12% (95% CI 10–15%)	13.2%
No atypia	7% (95% CI 4–10%)	CNB (14G) mean 3.9% (range 0–20%) VAB (11G) mean 7.7% (range 0–18.3%)
Atypia	32% (95% CI 23–41%)	CNB (14G) mean 46.7% (range 6.7–71.4%) VAB (11G) mean 33% (range 0–71%)
Radial Scar or Complex Sclerosing Lesions (RS/CSL)	8% (95% CI 6–11%)	
No atypia	6% (95% CI 2–13%)	CNB (14G) 9% (1–28%) VAB (11G) 1% (0–10%)
Atypia	18% (95% CI 8–32%)	CNB (14G) 33% VAB (11G) 2% (range 0–18%)

### Histopathology

Differentiating ADH from low-grade DCIS is challenging on histopathology and is often a diagnosis of exclusion, with cytological atypia and architectural changes similar to low-grade DCIS but with an involvement of no more than two membrane-bound spaces with an extent lower than 2 mm [18]. From the histological point of view, another lesion type that has to be considered is usual ductal hyperplasia. Usual ductal hyperplasia is characterized by the co-existence of more cellular types, not uniformly distributed, with formation of bridges, intercellular fenestration, and mixed-type cells proliferation without atypia. In this case, the expression of keratin with high molecular weight (i.e., CK5/6 and C14) can be useful to distinguish these cellular proliferations from the non-homogeneous monoclonal proliferation that characterizes ADH or low-grade DCIS [27, 28].

### Immunoprofile

Several studies have been performed to determine specific biomarkers for ADH, but currently no biomarker has been clinically validated. The limited data on the molecular biology of ADH demonstrates its close similarity to low-grade DCIS: Cells are positive for ER but negative for high molecular weight cytokeratins [18], and there is a low degree of chromosomic anomalies, with a consistent loss of 16q and gain of 1q [29]. The overlap of morphological and molecular characteristics further supports the hypothesis that ADH is a non-obligatory precursor of low-grade DCIS as well as invasive carcinoma.

### Prognosis

ADH is associated with a higher risk of invasive breast cancer, four to five times higher compared with the normal population [30]. In their study, Hartman et al. reported that of 698 women with either ADH (*n* = 330) or atypical lobular hyperplasia (*n* = 327) who later develop breast cancer, 69% of the women developed moderate or high-grade invasive ductal breast cancers; moreover, while the risk of developing breast cancer is elevated in both breasts, the risk is doubled in the ipsilateral breast compared with the contralateral breast [31].

### Diagnosis

**Fig. 1:**
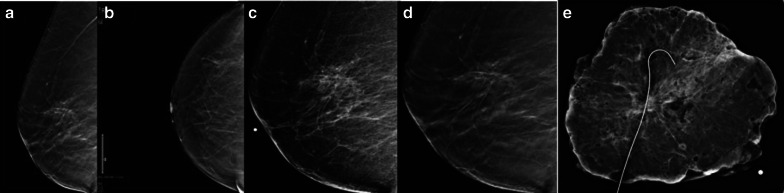
Full-field digital mammography (**a**) lateral medial oblique and (**b**) craniocaudal views and (**c**, **d**) 2D lateral focal spot compression and tomosynthesis showing a distortion in the upper outer quadrant, posterior third. **e** Surgical specimen; histology report confirmed atypical ductal hyperplasia with columnar cell changes and columnar cell hyperplasia

**Fig. 2:**
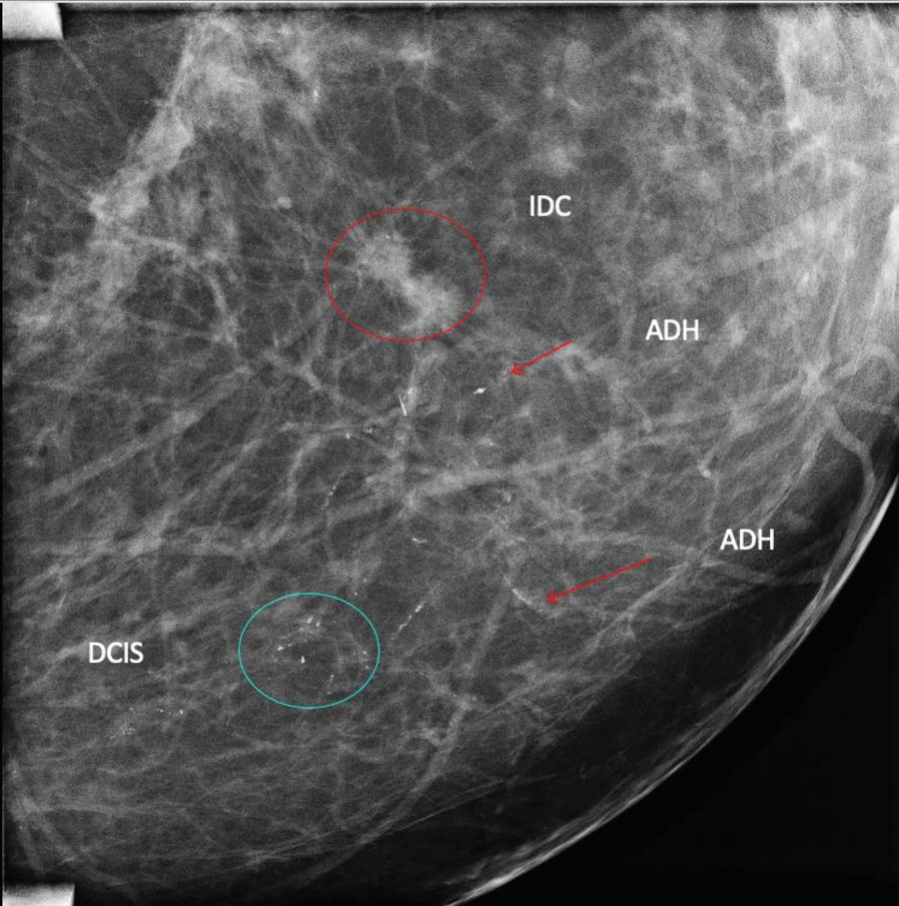
Digital mammography, lateral view with magnification of the left breast, showing two tiny spiculated masses (invasive ductal carcinoma (IDC)) associated with ductal calcifications (atypical ductal hyperplasia (ADH)) and distant group of pleomorphic calcifications (ductal carcinoma in situ (DCIS))

Imaging findings are not specific, and ADH can be found in the peripheries of a papillary lesion and even inside a fibroadenoma. At mammography, ADH can be seen as microcalcifications, mainly amorphous with grouped, linear, or regional distribution. It can also arise as a mass with or without microcalcifications. Only seldom is ADH detected at ultrasound. According to Mesurolle et al. [32], the sonographic appearance of ADH is non-specific but differs from invasive carcinoma; most ADH lesions appear as an irregular-shaped small hypoechoic non-mass or even mass lesion with micro-lobulated edges, no posterior acoustic features, and parallel orientation to breast parenchyma (Figs. 1, 2). Similarly, magnetic resonance imaging (MRI) features of ADH are non-specific; however, a non-mass enhancement is more frequent [33]. In Table 3, the most frequent imaging findings of ADH as well as the other type of high-risk lesions have been reported.

**Table 3:** Summary of imaging characteristics for the most common high-risk lesions

High-risk lesion	Imaging findings
Lobular Neoplasia (LN)	Amorphous microcalcifications and grouped distribution
Atypical Intraductal Epithelial Proliferation (AIDEP)	Fine pleomorphic microcalcifications and linear or segmental distribution Irregular-shaped small hypoechoic mass with micro-lobulated edges
Flat Epithelial Atypia (FEA)	Grouped microcalcifications
Papillary Lesion (PL)	A solid, round mass with well-defined borders; an intra-cystic mass; or a hypo-echoic, well-defined mass in a dilated duct With internal vascular stalk and with/without microcalcifications
Phyllodes Tumor (PT)	Ovoid high-density mass lesion with irregular edges and usually lobulated Large size
Radial Scars (RS)	Radiolucent architectural distortion with or without calcifications

### Management

ADH has the highest risk of underestimation among all types of high-risk lesions. The diagnosis of ADH at CNB or VAB is challenging, since ADH can be an isolated finding or a small component of DCIS [14]. Kohr et al. reported a high rate of underestimation with both CNB and VAB, although the latter is more accurate [34]. Schiaffino et al. [35] performed a meta-analysis including 93 studies and 6458 lesions in order to assess the risk of upgrade for ADH diagnosed through percutaneous procedures, according to the type of needle used for biopsy, imaging modality used as a guide for biopsy, and type of management (follow-up without surgery vs surgery). The results of this study showed that the upgrade rate was about 29% for lesions that underwent surgery vs 5% for those that underwent follow-up without surgery; however, the authors highlighted that the results are extremely heterogeneous, especially for lesions that underwent follow-up without surgery, and the heterogeneity can be explained mostly by the type of imaging modality and the caliber of the needle. Ko et al. [36] found that age > 50 years old, microcalcifications detected at imaging, size of the lesion, and multifocality were associated with a higher likelihood of upgrade to malignancy in a cohort of patients diagnosed with ADH at ultrasound-guided CNB and who subsequently underwent surgical excision. They proposed a scoring system based on clinical data (age, palpable vs non-palpable mass), pathologic analysis (focal vs non-focal ADH), and imaging features (microcalcifications at mammography, size) that resulted in a negative predictive value of 100%. However, their results should be interpreted with caution as the scoring system was evaluated in only 34 patients and needs to be validated in larger cohorts. When there are more than two foci of ADH on CNB and concurrent cellular necrosis, the latter is indicative (although not pathognomonic) of low-grade DCIS. Upgrade rates are also generally higher in biopsies with poor correlation between pathology and imaging.

Initially, the first International Consensus Conference on lesions of uncertain malignant potential (B3 lesions) [14] recommended follow-up without surgical intervention for all ADH lesions determined to have been completely removed through percutaneous biopsies. However, the upgrade rate for this type of lesions was found to be approximately 14% [6], which is too high for continuing with this initial recommendation. As such, the second International Consensus Conference replaced this initial recommendation with the recommendation that ADH management should be based on surgery and that clinical surveillance can only be justified in particular cases and after a proper multidisciplinary evaluation [5]. On the other hand, the NHSBSP (UK National Health Service Breast Screening Programme) suggests a more conservative approach, where in cases of a diagnosis of AIDEP following CNB or VAB, a VAE should be performed. The NHSBSP recommends follow-up in all those cases of ADH without atypia or if the lesion has been removed completely and only focal ADH with calcifications exists, while open surgery is recommended when, after a second assessment with VAE, atypia is found [11]. Both guidelines recommend cases to be discussed at a multidisciplinary team meeting, with radio-pathologic concordance, and dealt with on an individual basis.

If clinical surveillance is elected, in women diagnosed with ADH before age 40, there is a lack of consensus between guidelines on how to follow these patients. The majority consider these patients more than average risk for developing breast cancer and close imaging follow-up, even with MRI, should be considered [30, 37].

## Lobular neoplasia

Lobular neoplasia (LN) is considered both a risk indicator and a non-obligate precursor for breast cancer. LN is found in 0.5–4% of otherwise benign breast biopsies, and it can be seen at any age but is most frequent in premenopausal women [18, 38]. The lesion is multicentric in 85% of cases and bilateral in up to 67% of cases [39]. It is considered a risk factor for the subsequent development of invasive carcinoma in either breast [40].

### Histopathology

The term lobular neoplasia is used to represent atypical epithelial lesions characterized by the proliferation of poorly linked monomorphic cells due to the lack of E-cadherin expression, with possible pagetoid diffusion into galactophorous ducts. According to World Health Organization (WHO) classification, atypical lobular hyperplasia (ALH) and lobular carcinoma in situ (LCIS) represent the majority of LN and their differentiation is based on the extension of lobular units that are involved by the lesion: While LCIS compromises more than a half of TDLU acini, ALH is usually less extensive. Moreover, WHO classification encourages the use of the terminology “intraepithelial lobular neoplasia” (LIN), with a three-type classification (LIN1, LIN2, and LIN3). According to this classification, ALH is referred to as LIN1 and LCIS is referred to as LIN2, and LIN3 includes non-typical variants of LN which differ from the classic variant, either lacking the distension of the lobular unit (florid form) or lacking cellular atypia (pleomorphic form) [18, 40].

The prognostic significance towards malignancy for ALH and LCIS is not supported by evidence to date. Therefore, on both CNB and VAB samples, they are classified as B3 lesions, whereas pleomorphic or florid LN are classified as B5a (malignant) lesions [41]. LN may also be associated with other lesions including sclerosing adenoses, radial scars, papillary lesions, and fibroadenomas.

### Immunoprofile

Immunophenotyping and cytogenetic studies demonstrate similarities between LN and lobular invasive carcinoma as well as low-grade DCIS, namely high expression of estrogen receptor (ER) and progesterone receptor (PR), low Ki-67, and well-differentiated cytokeratin profile (CK5- and CK18+), and these findings lead some to consider LN as a precursor of invasive breast cancer [42]. However, two epidemiological cohort follow-up studies showed that the progression towards an invasive pattern is not compulsory [43, 44]. Therefore, the current consensus is to consider classic LN to be a risk marker [45] for breast cancer rather than a precursor of invasive breast cancer. On the contrary, the LCIS pleomorphic variant is negative for ER but is positive for human epidermal growth factor receptor 2 (HER2) as well as p53 and the proliferative index Ki67. For this reason, although epidemiologic studies have been not carried out, and also because of its strong association with invasive lobular carcinoma, the LCIS pleomorphic variant is generally considered a precursor of invasive breast cancer [45, 46].

The lack of expression of E-cadherin can be helpful in differentiating LCIS from DCIS or for classifying a lesion as indeterminate. Although rare, LCIS can express E-cadherin, and so in all cases where a histologic sample cannot be surely classified as LCIS or DCIS, the definition “carcinoma in situ with mixed ductal and lobular features” should be used [47].

### Prognosis

Molecular analysis has demonstrated that LN is a clonal neoplastic proliferation and a precursor to invasive cancer. The relative risk of developing breast cancer is 4–12 times higher for women diagnosed with LCIS compared with women without LN, and 4 to 5 times higher for women with ALH compared with women without LN. The upgrade rate after surgical excision ranges widely from 0 to 40% in the literature, which may be due to differences in inclusion and exclusion criteria used in different studies (Table 2). One main inclusion criterion should be pathologic–radiologic agreement, especially considering that research studies carried out over the last decade have demonstrated that poor agreement between pathologic and imaging findings are associated with higher upgrade rates [48, 49]. For example, Murray et al. demonstrated in 85 cases of classic LN that there was a significant difference in the upgrade rate among cases with and without radiologic–pathologic discrepancy (38% and 3%, respectively) [48].

A study by Genco et al. which evaluated the upgrade rate after surgical removal of classic LN showed that the upgrade rate was significantly higher for cases with previous or current breast cancer, radiologic asymmetry, and architectural distortion [50].

After the diagnosis of LN, the relative risk of developing cancer is 1–2% after one year, 15–17% after 15 years, and 35% after 35 years, with relatively equal rates of ipsi- and contralateral breast cancer (8.7 and 6.7%, respectively) [5, 33, 38]. The risk further doubles when the presence of proliferating high-risk lesions is accompanied by family history of breast cancer. Although all types of invasive carcinoma have been observed after a diagnosis of LN, invasive lobular carcinoma or special-type carcinomas are seen with higher frequency than in the general breast cancer population [51].

### Diagnosis

LN has no typical radiological or clinical manifestations; it is usually discovered fortuitously in breast tissue biopsied for other reasons [14], often as an incidental lesion in association with other radiological abnormalities [52, 53] and without any specific features according to the Breast Imaging Reporting and Data System (BI-RADS) lexicon [54].

**Fig. 3:**
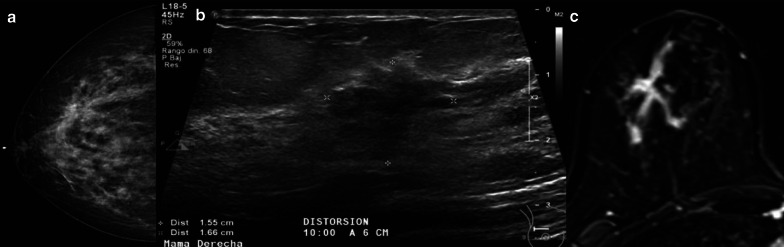
**a** Mammography (craniocaudal view) demonstrated hyperdense distortion in the upper outer quadrant. **b** Ultrasound shows a corresponding irregular and hypodense mass, 1.6 cm, at 10:00 o´clock. **c** The mass was confirmed on magnetic resonance imaging as a non-mass enhancing corresponding to classic lobular carcinoma in situ (10 cm) with microinvasion (< 1 mm)

On mammography, LN usually goes unnoticed, but when detected, it can be seen as microcalcifications, with amorphous morphology and grouped distribution [31, 38, 55]. In Rendi et al.’s study which included 106 cases of LN (73 ALH and 33 LCIS), imaging findings on mammography led to a biopsy in 74% of the cases [55]. Less frequently, LN can be seen as a mass, architectural distortion, or asymmetry (Fig. 3). On ultrasound, LN may appear as a shadowing, avascular, irregular, hypoechoic mass [51]. As LN generally does not present as a mass either on mammography or ultrasound, a diagnosis of LN generally does not sufficiently explain a mass on imaging. A diagnosis of LN in a BI-RADS category 5 lesion should also be considered a discordant result warranting surgical excision because of the high frequency of cancer at surgical excision in category 5 lesions [56]. On contrast-enhanced MRI, the evidence is limited, but some authors have suggested that LCIS may present as non-mass enhancement on MRI images [57] (Table 3).

### Management

Management of classic LN diagnosed on CNB remains controversial even though recent studies recommend conservative management based on the low upgrade rate reported [48, 58, 59]. Currently, the second International Consensus Conference on B3 lesions recommends that a lesion containing classical LN which is visible on imaging should undergo excision with VAB. Thereafter, surveillance is justified if there is no pathological–radiological discordance and no residual lesion. By contrast, morphological variants of LN that are classified as B5a lesions (LIN 3, pleomorphic LCIS, and florid LCIS) should undergo open excision [5]. The UK NHSBSCP is on the same page, suggesting VAE for second assessment after a diagnosis of LN at CNB or VAB. In cases of no additional LN, unifocal LN, radio-pathologic concordance, and there is no radiological suspicion of malignancy, imaging follow-up should be recommended by the multidisciplinary team. Surgery is considered in those cases of radio-pathological discordance or radiological suspicion of malignancy and in cases of upgrade to pleomorphic LCIS, DCIS, or invasive carcinoma at VAE [11].

Similar considerations for imaging follow-up of ADH have been made for LN. In women diagnosed with LN, close imaging follow-up with beast MRI should be considered, especially if other risk factors are present [30, 37].

## Flat atypical epithelia

Flat atypical epithelia (FEA), also known as columnar cell changes with atypia or cellular hyperplasia with atypia, is a non-malignant, atypical lesion of the breast. The WHO defines FEA as a neoplastic intraductal alteration of the breast characterized by replacement of native epithelial cells by a single layer or three to five layers of mildly atypical cells without architectural atypia [45]. Although several studies have been performed, the clinical meaning of FEA is still unclear. The incidence of pure FEA is approximately 1–2% of benign breast biopsies [20]. It is frequently discovered secondary to suspicious microcalcifications [60]. In addition, FEA is often found in association with other high-risk lesions of the breast, such as ALH, ADH, or LN, although currently it is increasingly referred to as a precursor of the latter alteration [61] and also considered, together with ADH and DCIS, as a non-obligatory precursor of invasive carcinoma [62, 63]. Moreover, FEA can co-occur in in situ or in invasive breast cancers, more typically tubular carcinoma [64].

### Histopathology

From the histological point of view, FEA appears as a neoplastic alteration in which normal luminal cells are replaced by one layer or three to five layers of columnar cells and the TDLU shows a low grade of atypia [45]. Cellular morphology can vary from the cuboid aspect to the columnar one, but usually bridges and micropapillary formations are absent, hence the name “flat.” The acini of involved TDLUs are variably distended from a secretory or floccular material with microcalcifications [18].

Generally, two forms of columnar metaplasia are distinguished: columnar cell changes, in which acini are lined by one or two layers of modified epithelial cells; and columnar cell hyperplasia, in which acini are lined by more than two layers of epithelial cells [65–67].

### Immunoprofile

Immuno-phenotype studies show a correlation between FEA, DCIS, and invasive breast cancer [38]. In fact, FEA cellular types are strongly and widely positive for ER and PR, and negative for low molecular weight cytokeratin, with a low Ki-67 index [18, 45]. In addition, cytogenetic studies of FEA lesions associated with DCIS and invasive carcinoma show similar changes in their genetic molecular profile [39]. For several authors, the continuum of phenotypical and cytogenetic lesions constitutes the basis for considering FEA to be a non-obligate precursor of low-grade DCIS and invasive carcinoma [62, 63, 68], in which FEA represents the first step on the carcinogenesis pathway.

### Prognosis

FEA is of great scientific interest as the morphological spectrum of intraductal proliferations from FEA to ADH and low-grade DCIS seems to represent a continuum. FEA seems to be associated with a very slight increased breast cancer risk (1–2 times), which is substantially lower than the risk due to ADH. While the breast cancer risk is only slightly elevated, it is important to note that this is a lesion that, by definition, contains a degree of epithelial atypia.

Forester et al. in their meta-analysis demonstrated that the upgrade rate to malignancy for FEA lesions is 11% [15]. Malignant findings after FEA diagnosed at CNB or VAB are usually in the form of ADH and low-grade DCIS, while invasive carcinoma (in most instances highly differentiated) can occur but less frequently [5]. No significant differences have been found in the underestimation rates of FEA according to the type of biopsy procedure and caliber of the needle used. For both CNB and VAB, underestimation rates have been estimated to be between 0 and 21%. In the literature, among FEA cases diagnosed at CNB, the underestimation rate generally varies from 10 to 21% [69–71]. Ceugnart et al. reported an underestimation rate of 6% from a retrospective series of 52 patients of FEA diagnosed on large-core biopsies with 11–8-G needles who then underwent surgery [72]. In a study by Villa et al. involving 121 FEA cases diagnosed at VAB, the underestimation rate of malignancy was 5.8%, without a statistically significant difference between the two calibers of needle used (9G and 11G); however, when comparing cases with and without residual microcalcifications within the 9G group and the 11G group, the underestimation rate was 0% for patients without residual microcalcifications in both groups and 18% and 16% for those with residual microcalcifications in the 9G group and 11G group, respectively [73].

### Diagnosis

**Fig. 4:**
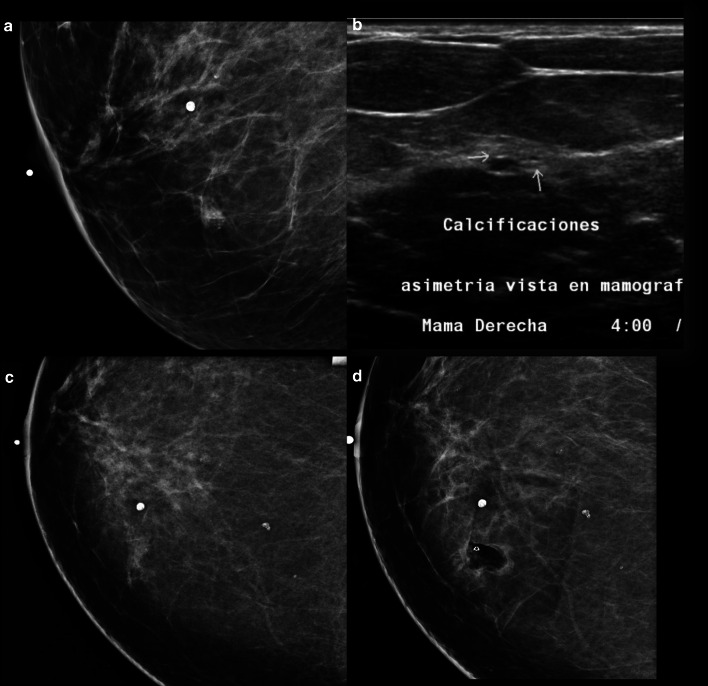
**a** Mammography (lateral medial oblique view) demonstrates a focal asymmetry with amorphous calcifications in the inferior inner quadrant. **b** Ultrasound shows a group of microcysts with punctiform and echogenic elements representing calcifications at 4:00 o´clock. **c**, **d** Stereotactic biopsy performed showing complete excision, with the metallic clip on site and no residual hematoma. Final Histology flat epithelial atypia

As these lesions produce intraluminal secretions, they commonly present mammographically as fine amorphous or branching microcalcifications with associated marked duct dilatation [9, 74] (Fig. 4). Data about the presentation of FEA on ultrasound are scarce, but FEA can be detected as a non-specific mass with or without microcalcifications. Up to now, no MRI morphologic or signal characteristics have been found to be helpful in the identification of FEA; however, the lack of enhancement with a high NPV, up to 100% of MRI can be helpful [74, 75].

### Management

Management for these lesions (surgical excision vs surveillance with radiological follow-up) remains controversial. Current guidelines increasingly favor surveillance if the lesion is small and radiological findings are completely removed by CNB or VAB [5].

According to AGO (German Gynecological Oncology Group) guidelines, when FEA is found at CNB or VAB, surgery can be avoided in the following situations: small lesion (< 2 TDLU involved at VAB) and/or partial or complete resolution at imaging [14]. On the contrary, surgical intervention is mandatory if the lesion is detected as a wide area of microcalcifications or in the case of discrepancy between pathology and radiology. If FEA is detected within surgical resection edges, no other treatment is recommended unless microcalcifications have not been completely removed. The second International Conference on B3 lesions established that a histologic sample with FEA detected at imaging should be obtained via VAB. Thereafter, close imaging follow-up is mandatory [5, 76, 77]. The NHSBSP recommends a similar approach in favor of VAE. Surgery should be considered only in cases of radio-pathological discordance, atypia or upgrade to DCIS or invasive cancer [11] (Table 1).

## Radial scar and complex sclerosing lesion

Radial scar (RS) is a breast lesion that may mimic invasive carcinoma because of its stellate configuration. RS is a focus smaller than 10 mm while complex sclerosing lesion (CSL) refers to a lesion which is larger than 10 mm and which has more complex features [14, 18, 78]. RS and CSL are classically non-palpable. [79]. Pathogenesis is unknown: Possible causes include localized inflammatory reaction and chronic ischemia with subsequent slow infarction [80]. Neither should be confused with otherwise explainable breast scars that occur after surgery, abscess, or blunt trauma. Radial scars are uncommon in women younger than 30 years and are seen most frequently in women 30–60 years old [81].

### Histopathology

**Fig. 5:**
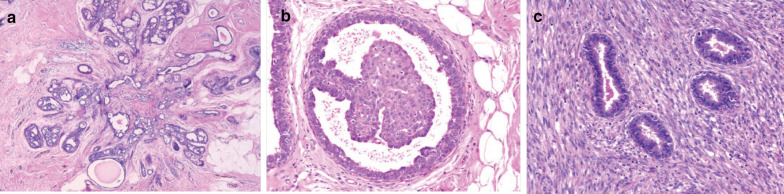
Histopathology representation of three B3 lesions. **a** Radial scar showing the typical stellate shape, central fibrosis, and some degree of epithelial proliferation. **b** Microscopic intraductal papilloma arising in a small, dilated duct. **c** A close view of benign phyllodes tumor showing tubular epithelial elements. Courtesy of Dr. Ramnani https://www.webpathology.com/

RS and CSL are characterized by a central fibroelastic core, which is surrounded by elastic fibers and which contain one to several ducts showing obliterated mastopathy (Fig. 5a). In addition, other ducts converge into the scar-like area in a stellate fashion. The epithelium lining the latter ducts may show a great variety of changes, the most frequent being benign epithelioid (usual ductal hyperplasia). The central scar-like area together with stellate appearance of the outer ducts easily mimics invasive carcinoma, both on radiological and histological grounds [5, 18, 78].

### Prognosis

RS and CSL are proliferative lesions which are frequently associated with other high-risk lesions and proliferative lesions such as atypia, and which may themselves contribute to the high frequency of associated cancers and to the upgrade rate to malignancy at excision. The prognosis of RS/CSL depends on the presence of associated atypia [78]. Previous studies have demonstrated that cancers are often found in the periphery of RS/CSLs and this eccentric location can lead to sampling error when performing CNB [82, 83]. The risk of subsequent breast cancer associated with RS found on pathology with and without atypia ranges from 1.1–3.0 to 2.8–6.7%, respectively [14]. If atypia is not present at histologic analysis, the chance of a malignant evolution is increased with architectural distortions, large size (> 10 mm), calcifications, and older age [84–86]. Recently published data suggest that in cases of RS diagnosed at CNB or VAB, accurate and detailed radiological–pathological correlations must be obtained, lesions < 10 mm have a lower rate of cancer upgrade, and histology is vital in the evaluation of presence or absence of atypical features within the lesion [5]. Patients with RS in benign breast biopsies have twice the risk of developing subsequent breast cancer [81].

### Diagnosis

**Fig. 6:**
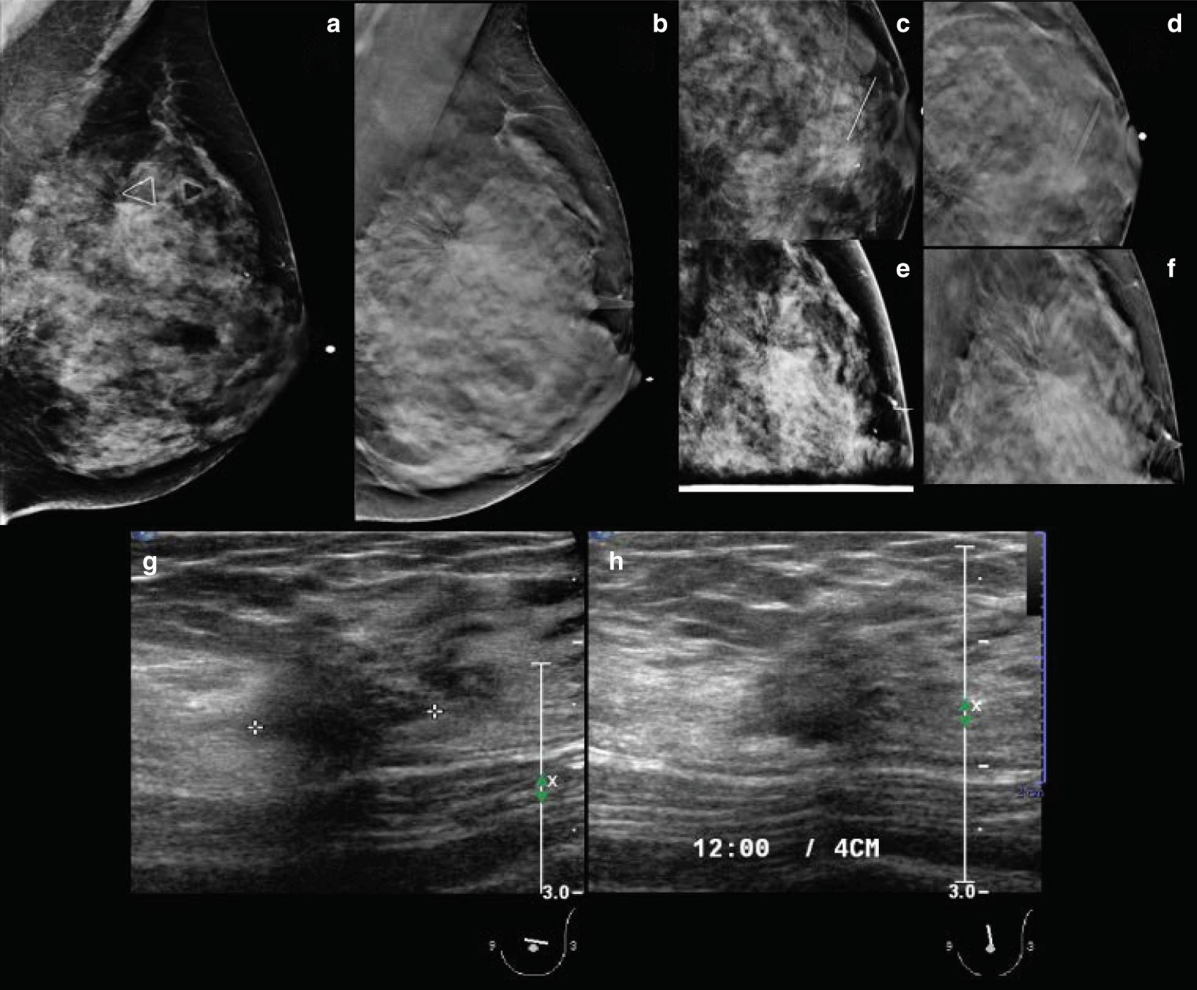
**a** Digital mammography (lateral medial oblique view), (**b**) tomosynthesis (lateral medial oblique view), (**c**) digital mammography (craniocaudal view), (**d**) tomosynthesis (craniocaudal view), (**e**) digital mammography (lateral view), and (**f**) tomosynthesis (lateral view) with focal compression of the left breast showing a palpable black architectural distortion with calcifications in the union of upper quadrant (12:00 o’clock), posterior third. **g**, **h** Diagnostic ultrasound. Final histology confirmed a radial scar

The mammographic appearance of a RS/CSL typically consists of a radiolucent star-shaped lesion, which can be confused with a form of presentation of invasive carcinoma. Over the years, authors have reported certain findings that may be helpful in detecting RS/CSL at mammography, including the presence of radiolucent linear structures paralleling the radiopaque spicules and the “black stars” appearance over the “white stars” appearance of breast cancer [80]. Other typical features are: changing distortion image on different mammographic projections, asymmetry, lack of a palpable mass, and thickening with attraction of the surrounding cutaneous planes [87]. Calcifications are a common finding; in some cases, they may be the sole suspicious finding leading to biopsy (Fig. 6).

Nowadays, the use of recently developed digital breast tomosynthesis techniques has gained momentum, due to their more accurate visualization, characterization, and identification of architectural distortion [88–91]. Architectural distortion on diagnostic digital breast tomosynthesis has been reported to demonstrate malignancy in over one-third of cases at pathology and also to demonstrate a high-risk lesion at core biopsy in another one-third of cases, with RS being the frequently reported histology among high-risk lesions [92].

With the increasing use of ultrasound in both screening and diagnostic settings, an increasing number of mammographically occult RS/CSLs have been detected at ultrasound and diagnosed at subsequent ultrasound-guided CNB, ranging from approximately 15.7 to 39% among radial scars without atypia diagnosed at percutaneous biopsy [93]. However, ultrasound findings pathognomonic for RS/CSL and for their differentiation from malignancies have not yet been discovered [94]. The sonographic appearance of RS is variable, ranging from no clear correlate to a hypoechoic, irregular mass with indistinct margins, or a focal area of shadowing with no associated mass [94, 95]. In some cases, RS may show at elastography as inherent stiffness.

The MRI appearance of RS/CSL is similarly variable. In Ha et al.’s study involving 13 radial scars that were imaged with MRI, six lesions were not detected, six appeared as a mass (three with irregular margins, four with a homogenous enhancement pattern, and five with a Type 3 enhancement kinetic curve), and one appeared as non-mass enhancement with regional, homogeneous enhancement, and a Type 3 enhancement kinetic curve [96] (Table 3).

### Management

RS/CSLs are only rarely associated with atypia or DCIS. Therefore, RS lesions are most often benign lesions, and recent studies with careful radiological correlation have indicated that open surgical excision may not be necessary for small lesions and complete removal of the imaging abnormality. The current recommendation from the second International Consensus Conference on B3 lesions is that a RS/CSL lesion, which is visible on imaging, should undergo therapeutic excision with VAE [5]. When RS is associated with atypia (such as FEA, ADH, or classical lobular neoplasia), management is the same as recommended for cases of atypia alone; therefore, surgery should be performed [5, 41]. A similar approach is shared by the NHSBSP, where VAE is recommended if no atypia is found, with no need for imaging follow-up, whereas in cases of RS associated with atypia or upgrade to DCIS or invasive carcinoma, surgery is recommended [11] (Table 1).

## Papillary lesions

Papillary lesions include a large number of rare lesions, accounting for approximately 3% of all breast malignancies [59]. They are seen most frequently in women aged 30–50 years old; by contrast, they are rarely reported in adolescents [18]. Papillary lesions may be classified as solitary intraductal papillomas, multiple intraductal papillomas, papillomas with atypia, papillomas with DCIS, and papillary invasive carcinomas [97]. Their appearances in the breast vary clinically, radiologically, and pathologically, and familiarity with the features of malignant and benign papillary lesions is required to plan management.

### Histopathology

Papillary lesions are composed of papillary fronds which are attached to the inner mammary ductal wall through a fibrovascular core and which extend into the lumen of the duct (Fig. 5b). Ductal epithelial and myoepithelial cells line the fibrovascular core, and it is these epithelial cells that can undergo degrees of apocrine metaplasia [98]. From the histological point of view, papillary lesions are an inhomogeneous group of lesions, classified by the WHO as follows:

- Papilloma without atypia
- Papilloma with atypia (ADH or classical LN), both belonging to the B3 category on BI-RADS (small solitary papillomas (< 2 mm) can be categorized as B2 lesion, if the lesion is completely surrounded by a duct structure)
- Papilloma with DCIS or papilloma completely involved by more extended DCIS (encapsulated papillary carcinoma)
- Solid papillary carcinoma belonging to B4 or B5a category [18]

Usually, papillary lesions can be divided according to their localization into two main groups: central (generally isolated), i.e., arising from the ducts of the sub-areolar region sparing TDLU; and peripheral (generally multiple), i.e., originating from TDLU following the involvement of galactophorous ducts [99].

### Prognosis

The risk of an invasive cancer onset is two times that of the normal population for the central form and three times for the peripheral form, with a higher risk of invasive cancer onset of up to 7.5% in the case of papilloma with atypia [18]. The diagnosis of multiple peripheral papillomas (so-called “papillomatosis”) at CNB increases the risk of an invasive breast carcinoma or DCIS up to 30% (if atypia is present).

It is important to note that the upgrade rates for papillary lesions vary widely in the literature and may be as low as 3.1% for solitary papillomas [100]. Several studies have attempted to identify which patients among those with papillary lesions are suitable for surgery, focusing on clinical, radiological, and histological features that can predict the upgrade rate after percutaneous biopsy [101–104]. In their study, Qaemali et al. investigated whether histologic findings (necrotic foci and calcifications) or clinical data (age, symptoms) could predict malignancy, demonstrating that none of those factors achieved a statistical significance for that purpose [81]. On the contrary, other researchers have found that microcalcifications (identified histologically), lesion size (at least > 1 cm), older patient age, and certain histologic features are among the factors that are most predictive of upgrade to malignancy [105–107]. Khan et al. evaluated the rate of concurrent malignancies for intraductal papilloma diagnosed at CNB and assessed the long-term risk of developing a breast cancer, obtaining an upgrade after surgery of 7.5% for intraductal papillomas that were atypia-free vs 32% for those with atypia and 33% for patients with simultaneous ADH/ALH [100]. Other studies focused on the type of needle used, which resulted in an upgrade rate ranging from 0 to 12% for papillomas without atypia when a CNB is performed [99, 108], while no upgrade to malignancy has been reported for papillomas without atypia diagnosed with VAB [85, 109, 110]. For papillomas with atypia, the upgrade rates to malignancy are more variable, ranging from 21 to 72% for diagnosis at CNB and from 0 to 28% for diagnosis at VAB [86, 111] (Table 2).

### Diagnosis

**Fig. 7:**
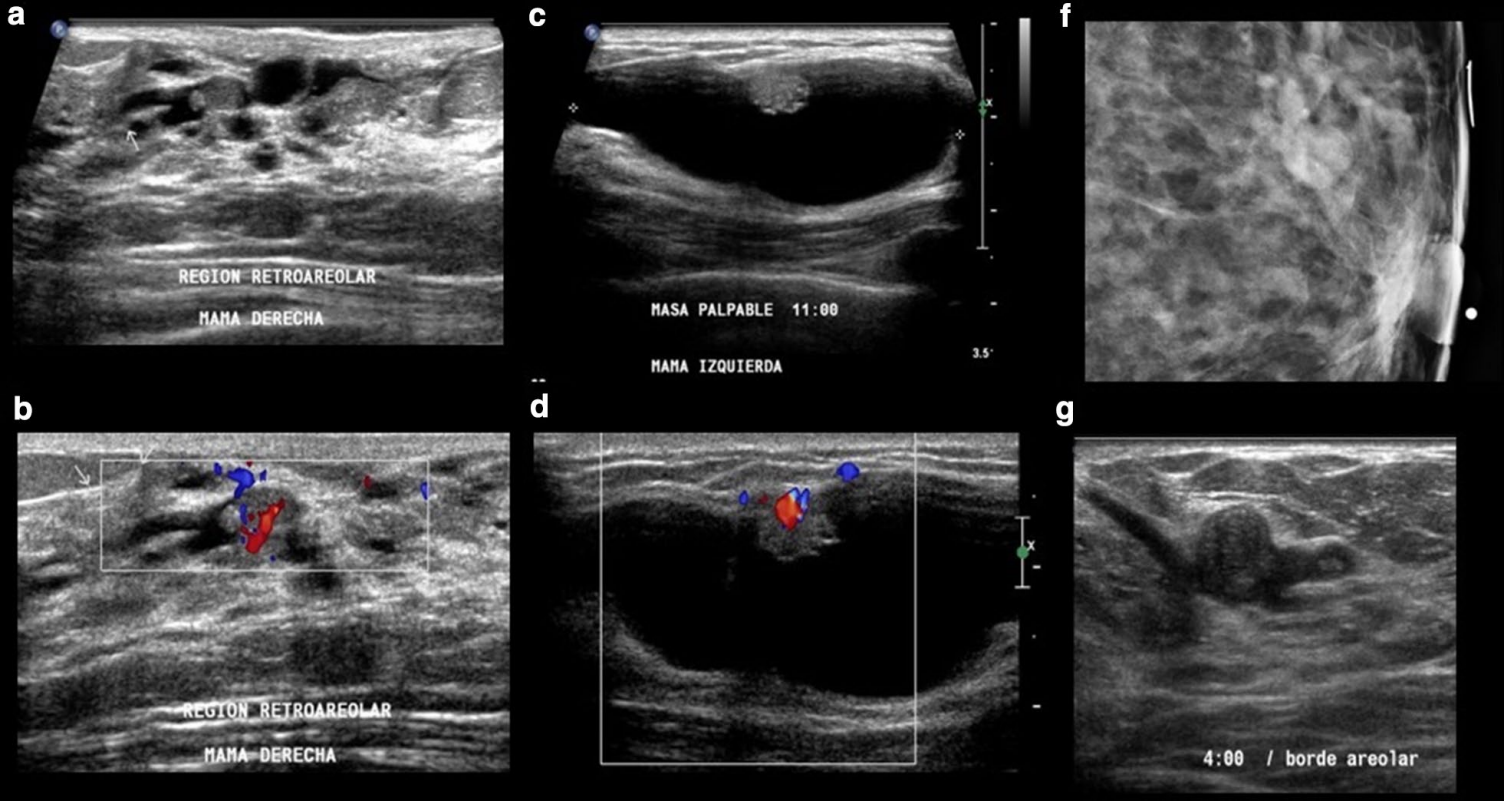
Three different cases of solitary papillomas. 1. **a**, **b** Ultrasound shows dilated ducts and an oval and isoechoic intraductal mass with a fibrovascular stem, demonstrated with color Doppler imaging. 2. **c**, **d** Ultrasound shows a complex cystic mass with dominant cystic component within a small solid nodule. 3. **e** Mammography demonstrates a lobular isodense mass in the retroareolar region and (**f**) ultrasound shows a corresponding intraductal mass

Papillomas can be asymptomatic and detected at routine breast screening. However, when symptomatic, they can be present as a palpable mass near the nipple or with bloody nipple discharge, in up to 20–50% of cases, due to pedicle torsion [34, 91]. Mammography is limited with poor sensitivity and specificity; when visible, mammographic findings are represented by asymmetric areas of increased density, dilated ducts, or solitary lesions with associated microcalcifications. Since the features of papillary lesion at mammography can be non-specific, the main role of this technique is the detection of microcalcifications that can be suspicious and related to papillary malignant lesions [92] (Fig. 7).

Breast ultrasound is of great value in the diagnosis of papillomas, whether conventional ultrasound, Doppler ultrasound, or elastography [112]. Ultrasound may demonstrate a solid, round mass; a mass with well-defined borders; an intra-cystic mass; or a hypo-echoic, well-defined mass in a dilated duct. The fibrovascular stalk of papillomas can be further assessed with color or power Doppler ultrasound. Elastography can show increased stiffness of the area within the duct which increases the degree of suspicion of malignancy, which can be helpful in differential diagnosis [113].

MRI is useful for determining the extent of disease in cases when a diagnosis of papilloma has already been established. MRI allows a comprehensive assessment including the identification of connections with the ductal system and a more accurate estimation of disease extent in cases of malignant papillary lesions. The MRI protocol should include acquisitions before and after intravenous contrast administration, due to the significant enhancement of small papillary lesions. MR ductography images can also be obtained to map the entire breast ductal system, whereby the ductal system would be characterized by strong enhancement in which papillomas can be detected as defects [114] (Table 3).

### Management

Certain clinical and imaging factors including patient age, lesion multiplicity, and peripheral location are associated with increased breast cancer risk. Overall, management depends on a number of factors, including age at diagnosis, lesion type, and presence of atypia. As previously mentioned, it is well established that a papillary lesion with atypia diagnosed at CNB can show malignant evolvement or simultaneous malignancy [100, 101]. The histopathological assessment of papillary lesions with atypia requires the pathologist to assess the size of the area of atypia which cannot be measured if it is distributed between multiple cores. Papilloma with atypia should undergo surgical excision, according to NHSBSP [11]. For papillomas without atypia, management options are variable. A papillary lesion which has already undergone CNB or VAB does not need to undergo further surgical excision if the histologic sample is sufficient (100 mm^2^) and an agreement with imaging findings is found; for papillomatosis, surgery is recommended [5, 115]. Current recommendations from the second International Consensus Conference on lesions of uncertain malignant potential (B3) is that a papillary lesion that is visible on imaging should undergo VAE. For larger lesions that cannot be completely removed, surgery is recommended. The NHSBSP suggests VAE as a second assessment tool in cases where a papillary lesion without atypia is diagnosed at VAB or CNB [11] (Table 1).

## Bland-looking spindle cell lesions

Bland-looking spindle cell lesions encompass a heterogenous group of tumor-like and tumor entities, ranging from benign reactive to low-grade malignant neoplasms with metastatic potential [116]. The more common spindle cell lesions encountered in the breast are spindle cell metaplastic carcinoma, which is a malignant lesion; myofibroblastoma, pseudoangiomatous stromal hyperplasia (PASH), and fibromatosis, which are benign lesions; and phyllodes tumor, which is consider histologically a B3 lesion [117]. As for the differential diagnosis of spindle cell lesions, these lesions show considerable morphologic and immunophenotypic overlap. As such, definitive classification of a spindle cell lesion may not be possible with the limited sampling of a CNB where defining histologic features may not be present. A targeted immunohistochemical panel is frequently needed to fully evaluate and classify spindle cell lesions of the breast. For the purpose of this review which focuses on B3 lesions, we will go into details for only phyllodes tumors.

## Phyllodes tumor

Phyllodes tumor of the breast is a rare tumor that accounts for fewer than 1% of all breast tumors and 2–3% of fibroepithelial neoplasms [18, 118]. Although it is often characterized by a large size and/or rapid growth, it can exhibit a slow-growth pattern [97]. Commonly, this tumor occurs in women aged 40–51 years, although in Asian countries, the average age of occurrence is about 25–30 years [119].

### Histopathology

Phyllodes tumors are rare breast fibroepithelial lesions and are similar to fibroadenomas and hamartomas, and they can range from benign to borderline or clearly malignant forms from the histological point of view (Fig. 5c). In some cases, the distinction between the typical fibroadenoma and phyllodes tumor can be challenging.

Therefore, WHO recommends the diagnosis of fibroepithelial tumors (BI-RADS 3 category) for unclear cases. Briefly, both benign and borderline phyllodes tumors are considered B3 lesions of the breast, histological classification, whereas malignant Phyllodes tumors are included into the B5b category [18]. B3 lesions, especially benign phyllodes tumors, are the most frequently detected, while only 20% of all are borderline or malignant [27].

### Prognosis

The majority of phyllodes tumors have a benign behavior, with a risk of recurrence of approximately 10–20% for benign lesions, and up to 30% for borderline or malignant lesions. Metastatic capacity depends on histology, with 15–20% for malignant phyllodes tumors [5, 120, 121]. Several studies also confirmed that involved margins are a strong predictor for recurrence [122, 123].

### Diagnosis

The typical appearance of a phyllodes tumor is a unilateral mass that is frequently symptomatic (palpable) and mobile (not fixed to deep tissues); as a large palpable tumor, it stretches the skin with distension of superficial vein, with a size that ranges from 2 to 5 cm [18, 124]. Imaging features of phyllodes tumors usually overlap with more common imaging features of fibroadenomas, with rapid growth as a pathognomonic characteristic for phyllodes tumor.

**Fig. 8:**
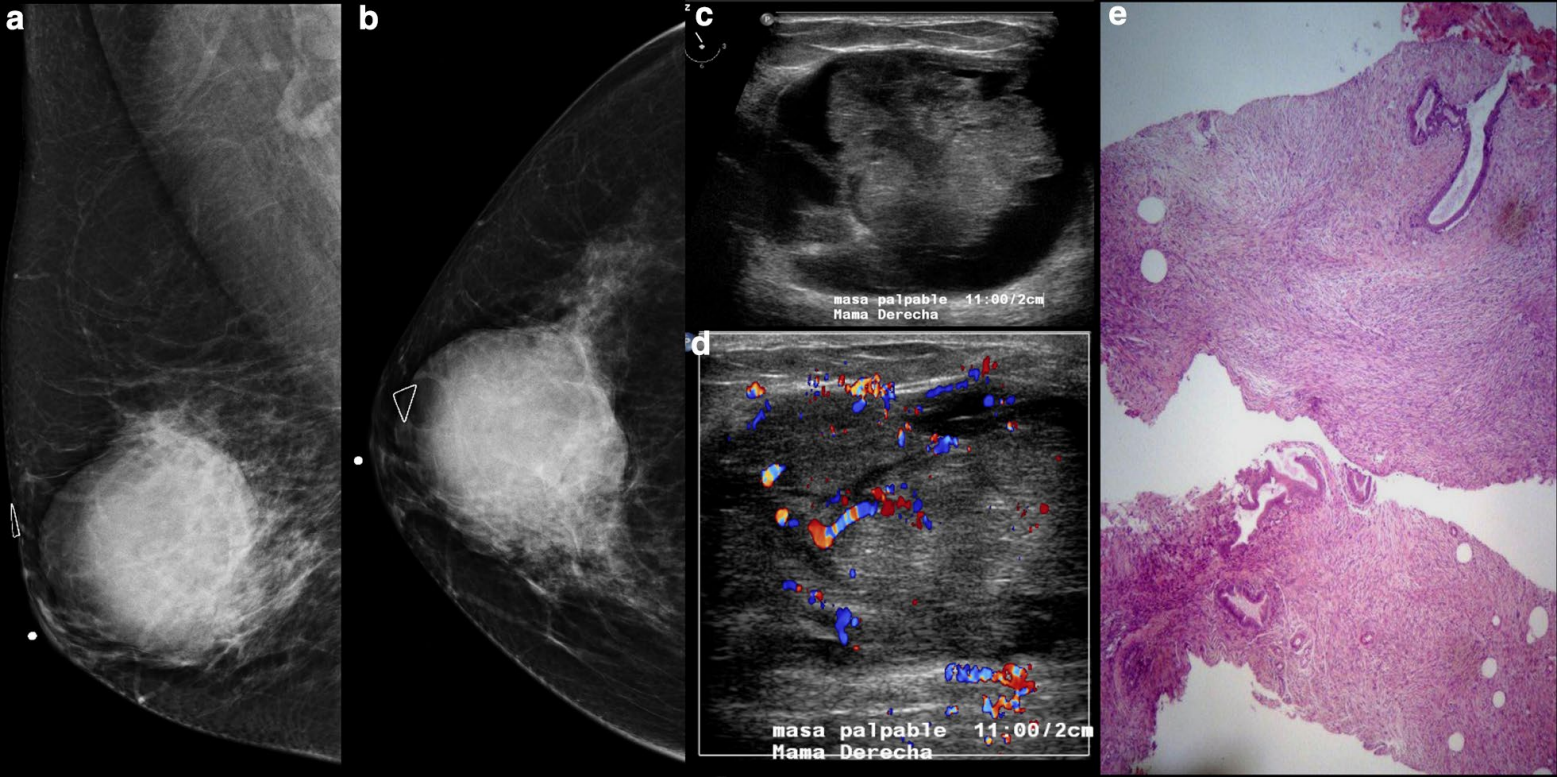
**a**, **b** Conventional views of digital mammography of the right breast demonstrate a round and hyperdense mass. Ultrasound (**c**, **d**) demonstrates a corresponding complex mass with inner vascular flow with color Doppler imaging. **e** Histology image with the diagnosis of borderline phyllodes tumor (low-grade sarcoma)

At mammography, phyllodes tumors appear as a big ovoid high-opacity mass with irregular edges and usually lobulated [125]; coarse calcifications can be detected only rarely due to their fast growth, whereas it is possible to find fat content as a lucent halo of the margins. Therefore, digital breast tomosynthesis can be helpful, since it can allow a more accurate detection of such a component [106] (Fig. 8).

Ultrasound findings of phyllodes are variable. Several reviews report that these lesions can have either circumscribed as well as non-circumscribed borders [125, 126]; the most common characteristics are mildly hypoechoic internal echoes, a heterogeneous internal echo pattern, and a posterior acoustic shadow [126, 127], with the latter presenting frequently in malignant variants. Cole-Beuglet et al. reported an intramural cyst in a phyllodes tumor. They proposed that the diagnosis of phyllodes tumor should be considered if intramural cysts suggest focal necrosis or degeneration within a large, lobulated, solid breast mass [128].

There is lack of data regarding the assessment of phyllodes using MRI. Several authors focused on identifying signal features that may predict benignity or malignancy. Farria et al. reported that rapid enhancement after gadolinium injection is associated with benign phyllodes tumor [129]; in addition, Kinoshita et al. reported that smooth margins, lobular or polygonal shape, and occasional cystic changes or internal septa are characteristic of benign phyllodes tumor [130]. In Yabuuchi et al.’s study, which evaluated MRI characteristics in relation to phyllodes histologic grade, cystic change with irregular wall, tumor signal intensity higher than normal breast tissue signal intensity on T1-weighted images, tumor signal intensity lower than or equal to normal breast tissue signal intensity on T2-weighted images, and/or low ADC on diffusion-weighted images, were found to be suggestive of histopathologically malignant phyllodes tumor of the breast [131].

### Management

Available data suggest that the most common management for phyllodes tumor is surgical excision [5, 132]. The possibility of phyllodes underestimation diagnosed at CNB is approximately 20%, whereas it is lower at 8% when diagnosed at VAB [133]. Based on the current evidence, the second International Consensus Conference on lesions of uncertain malignant potential (B3) recommends that a phyllodes tumor, when found at CNB, should undergo open surgical excision with clear margins. If accidentally found at VAB without any corresponding imaging finding, surveillance of a benign phyllodes tumor is justified, while borderline and malignant phyllodes tumors require re-excision to obtain clear margins [5]. Similar guidelines are issued by the NHSBSP which recommend surgical excision for phyllodes. In case of rapid changes in size of a lesion previously biopsied as fibroadenoma, surgical excision should be performed [11] (Table 1).

## Conclusion

Diagnosis of high-risk lesions occurs frequently and incidentally at percutaneous biopsy, following suspicious imaging findings such as distortion, a group of microcalcifications, or even a mass. Traditionally, high-risk lesions are managed with surgical excision because of their low but latent potential of upgrade to malignancy. The most recent guidelines recommend a more conservative approach, sparing open surgery when this can be avoided. This trend has been adopted for the continued concerns raised regarding potential harms associated with unnecessary biopsies and surgeries when suspicious imaging findings are found. Further, radiological follow-up by means of mammography and breast MRI should be considered in patients diagnosed with high-risk lesions as they are not only non-obligate precursors of malignancy but also risk indicators for the homolateral and contralateral breast. It is essential that management of high-risk lesions takes on a multidisciplinary approach with close communication between all members of the breast team (surgeon, pathologist, oncologist, geneticist, radiotherapist) to assess the individual risk of patients with such a diagnosis and their suitability for either surgical excision or imaging surveillance.

## Data Availability

Not applicable.

## Abbreviations

ADH: atypical ductal hyperplasia
AIDEP: atypical intraductal epithelial proliferation
ALH: atypical lobular hyperplasia
BI-RADS: breast imaging reporting and data system
CNB: core-needle biopsy
CSL: complex sclerosing lesion
DCIS: ductal carcinoma in situ
ER: estrogen receptor
FEA: flat atypical epithelia
HER2: human epidermal growth factor receptor 2
LCIS: lobular carcinoma in situ
LN: lobular neoplasia
MRI: magnetic resonance imaging
PR: progesterone receptor
PT: phyllodes tumor
RS: radial scar
TLDU: terminal ductal-lobular unit
VAB: vacuum-assisted biopsy
WHO: World Health Organization
